# Thyroid Function Abnormalities in the Acute Phase of COVID-19: A Cross-Sectional Hospital-Based Study From North India

**DOI:** 10.7759/cureus.24942

**Published:** 2022-05-12

**Authors:** Yashendra Sethi, Nidhi Uniyal, Sonam Maheshwari, Richa Sinha, Ashish Goel

**Affiliations:** 1 Department of Medicine, Government Doon Medical College, Dehradun, IND; 2 Department of Community Medicine, Government Doon Medical College, Dehradun, IND; 3 Department of Physiology, Government Doon Medical College, Dehradun, IND

**Keywords:** t4, t3, tft, viral illness, covid-19, thyroid

## Abstract

Introduction

Viral illnesses like mumps, cytomegalovirus (CMV), and Cocksakievirus have been shown to affect the endocrine system, specifically the thyroid as a product of their systemic inflammatory process. The thyroid gland, having high levels of angiotensin-converting enzyme 2 (ACE2) is also predisposed to dysfunction due to coronavirus disease 2019 (COVID-19).

Methodology

A cross-sectional study was conducted using retrospective data of thyroid function tests in patients with COVID-19.

Results

The majority of patients with COVID-19 had normal thyroid function while low serum T3, seen in 47.3% of patients with severe disease, stood out as the most common thyroid abnormality in the acute phase of the disease. The disease severity was seen to correlate with the extent of thyroid function abnormalities, with severely diseased patients having lower T3 values and normal to low thyroid-stimulating hormone (TSH) values. Furthermore, a significant negative correlation was seen between TSH and the bio-inflammatory marker, C-reactive protein (CRP).

Conclusion

The acute phase of COVID-19 affects thyroid function in direct correlation with the severity of the disease.

## Introduction

Coronavirus disease 2019 (COVID-19) began as a pandemic in 2019 and showed a variety of unexpected effects on various systems of the body apart from the majorly affected respiratory system. It was declared a public health emergency of international concern (PHEIC) by WHO on January 30, 2020 [1].

Viral infections have been seen as notorious for affecting more than one system of body function as a result of their systemic inflammatory process. Several viruses have been shown in a catena of research to be associated with dysfunctions in the thyroid. Subacute thyroiditis has been reported during outbreaks of various viral infections, including mumps virus, cytomegalovirus, enterovirus, Coxsackievirus, hepatitis virus, and HIV [2-4]. Subacute thyroiditis is usually preceded by an upper respiratory tract infection. Patients having subacute thyroiditis in a mumps epidemic were shown to have circulating anti-mumps antibodies even without signs and symptoms of mumps [5]. Subacute thyroiditis has also been seen associated with COVID-19 [6]. There has been clear evidence of derangement of thyroid function in acute phases of some viral infections like HepB [7].

A significant interplay has been observed between the systemic inflammatory process by the severe acute respiratory syndrome coronavirus 2 (SARS-CoV-2) virus causing COVID-19 and thyroid function. SARS-CoV-2 acts by using angiotensin-converting enzyme 2 (ACE2) combined with transmembrane protease serine 2 (TMPRSS2) as the key molecular complex to enter the host cells. Interestingly, the expression of ACE2 and TMPRSS2 in the thyroid gland is even higher than in the lungs. The ACE2 receptor also plays a role in viral replication inside the cell [8]. The thyroid gland and the entire hypothalamic-pituitary-thyroid (HPT) axis are relevant targets of damage by SARS-CoV-2. Another possibility is central hypothyroidism induced by hypophysitis or by hypothalamic dysfunction, which is evident from low serum triiodothyronine and thyroxine levels associated with decreased TSH concentration [9-10].

Most patients with COVID-19 have been seen to be euthyroid with others having mild reductions in thyroid-stimulating hormone (TSH) and FT4 in keeping with a nonthyroidal illness syndrome [11]. But the pattern has shown variations and varies with the phase of the disease. Even SARS-CoV-2-induced olfactory dysfunction has been co-related with thyroid mechanisms and is triggered by a direct viral insult of both the olfactory nerve and the thyroid gland. The absence or delayed recovery of olfaction may be produced as a product of virus-induced downregulation of thyroid function, which may blunt the effects of thyroid hormones on the maturation and regeneration of olfactory neuronal cells [12].

It is well-known that the thyroid hormone demonstrates its effects on the immune system at both nuclear and cellular levels. The effects at the nuclear level occur by activating transcription factors aiding intracellular signaling and at cellular levels by modulating cytokine release on multiple cells [13-14]. Several studies have shown decreased T3 levels in the acute phase of critically ill patients [13,15].

There is a lack of concrete literature pointing to the expected effects of SARS-CoV-2 on thyroid function during the acute phase of disease correlating them with disease severity. The currently available literature points to both ends of the spectrum [16-18]. A recent meta-analysis has shown that abnormal thyroid function was associated with a higher risk of poor outcomes in COVID-19. Furthermore, it concluded that patients with thyroid abnormalities have shown a significantly increased risk of higher disease severity, ICU admission, mortality, and hospitalization [19]. No attempt so far has been made to correlate clinically assessed severity in the acute phase of the disease and the prevalence of thyroid abnormalities in each group. To add to the same, no conclusive study has yet been done in India to assess thyroid abnormalities in COVID, making a missing note in current data. This study aims to present an attempt to fill this gap in the literature and hence contribute to a better understanding of the disease. These may not only serve to add to the knowledge of the disease process but also serve as a background to guide methods for the treatment and management of such outbreaks, especially from potent agents of the same family of viruses. The study was done with the objectives of deciphering the effect of the acute phase of COVID-19 on thyroid function and the effect of the severity of disease of COVID-19 on thyroid function and elucidating the association of the bio-inflammatory marker, C-reactive protein (CRP) with the thyroid function test (TFT).

## Materials and methods

A cross-sectional, hospital-based study was conducted at the Department of Medicine, Government Doon Medical College, Dehradun.

Sample size

The sample size was: 57 (19+19+19) - Mild, Moderate and Severe COVID-19 infection. The sample size calculation is based on the mean difference (0.5) of TSH between Moderate and Severe COVID-19 patients reported in a recent study by Chen M et al. [20]. This sample size uses a 99% Confidence Interval with 90% power.

$n = (Z\alpha /2 + Z \beta )^{2} * 2 \delta ^{2} / d^{2}$

Where $Z\alpha /2$ is the critical value of the normal distribution at $\alpha /2$, $Z \beta$ is the critical value of the normal distribution at β, $\delta ^{2}$​​ is the population variance and d is the difference.

The grading of severity was done in accordance with Indian Council of Medical Research (ICMR) guidelines, taking patients with oxygen saturation (SpO2) at room air above 94% as mild cases, between <=94% and 90% as moderate cases, and <90 % as severe cases.

Data collection

Data were collected from hospital records of COVID patients in the Department of Medicine, Government Doon Medical College (GDMC), in the time period of March 2020 to September 2021. The data for thyroid function were taken from the time of presentation.

Inclusion Criterion

All admitted COVID-19 reverse transcription-polymerase chain reaction (RT-PCR) positive patients above the age of 18 years from the period from March 2020 to September 2021 were included.

Exclusion Criteria

Patients with deranged renal function or acute kidney injury (AKI), secondary bacterial infection (procalcitonin > 0.5), or diagnosed thyroid illness at the time of presentation were excluded, as these may have an independent effect on thyroid function.

Statistical analysis

Statistical procedures were performed using SPSS v.22.0 (IBM Corp., Armonk, NY). The normality of the distribution was tested using the Shapiro-Wilk test. T3, T4, and TSH levels and CRP did not follow a normal distribution. Inter-group differences were analyzed using the Kruskal-Wallis test. Associations were tested by Spearman’s correlation coefficient and a correlation matrix was created. The significance level was set at p>0.05.

Ethics approval and consent to participate

The study was approved by the Institutional Ethics Committee of the Government Doon Medical College with the reference number GDMC/IEC/2022/09. The study was based on retrospective data from hospital records of patients.

## Results

The mean age of our sample was 47.1 years and the distribution between genders was males - (Mild 11, Moderate 13, Severe 15) and females - (Mild 8, Moderate 6, Severe 4).

Our study showed that in the total sample of patients with COVID-19, 28% had raised T4 and around 9% had decreased TSH. Among mild disease patients, most patients had normal T3 (95%) and TSH (78.9%). Furthermore, 31.6% had raised T4. Among Moderate disease patients, most patients had normal T3 (84.2%) and TSH (84.2%). Also, 26.3 % had raised T4. Among severe diseases, most patients had normal TSH (84.2%). Interestingly, T3 was decreased in 47.3% of patients while T4 was raised in 26.3%.

Spearman's correlation was used to examine the correlation between various variables for the study; the following findings were found statistically significant:

Positive correlation - Mild - T3, T4 (r=0.486*); Moderate - T3, T4 (r=0.646); Severe - T3, T4 (r = 0.692)

Negative correlation - Moderate - TSH and CRP (r=-0.893), Severe - TSH and CRP (r=-0.541)

A positive correlation was found between T3 and T4, which became more pronounced with increasing disease severity; the correlation coefficient was highly increased between mild and moderate groups while the increase was just minimal for moderate to severe and a negative correlation was found between TSH and CRP, which became more pronounced between mild and moderate disease severity while it was less pronounced between moderate and severe disease severity.

A Kruskal Wallis test reported a statistically significant difference in the values of T3, T4, and CRP across the mild, moderate, and severe disease severity groups. T3 (H = 11.98, p =0.02) and T4 (H = 6.71, p = 0.035) were lower in higher disease severity while the median values of TSH also decreased with disease severity but the difference wasn’t statistically significant. The median values of CRP (H = 8.63, p = 0.013) were found to be higher in severe disease than in mild or moderate disease.

## Discussion

COVID-19, in congruence with many other viral illnesses like mumps, cytomegalovirus, etc., has been seen to cause thyroid abnormalities owing to its pathogenesis based on ACE2 and TMPRSS2 expression [8]. Furthermore, the thyroid hormone and immune system are closely related, and the effect of the thyroid on the immune system has been widely studied. The abnormal release of thyroid hormone affects the capability to mount an immunological response. It has been well-established that during the acute phase of the disease, the body overproduces cytokines and chemokines triggered by the SARS-CoV-2 infection [21]. In the case of the prolonged acute phase, there can be suppression of the thyroid axis, which can lead to a poor prognosis in patients with thyroid abnormalities [22]. Also, the majority of hospitalized patients with a critical illness are known to undergo transient thyroid hormone dysregulations [23-24] which is often termed nonthyroidal illness syndrome or TACITUS (thyroid allostasis in critical illness, tumors, uremia, and starvation) [25].

The acute phase of COVID-19 and thyroid function

In our study, the median values of TFT (Table 1) suggested that the most common cluster was euthyroid patients, but around 47.3% of patients with severe disease showed low serum T3 levels while around 15.8% with severe disease had low TSH, which may be indicative of secondary hypothyroidism (Table 2). This is in line with the findings of Lania et al., who found that around 5.2% of patients in their study were hypothyroid [26], and Muller et al., who found that a substantial proportion of patients with COVID-19, requiring a high intensity of care, present with thyrotoxicosis and low serum TSH concentrations [16].

**Table 1:** Median (IQR) values of various biochemical test reports of study subjects The median values suggested that most patients presenting with COVID-19 had TFT within the clinically acceptable normal range. IQR: interquartile range; TFT: thyroid function test; TSH: thyroid-simulating hormone; CRP: C-reactive protein; IL-6: interleukin 6

PARAMETER	MEDIAN IQR (Q1-Q3)
Total T3	0.99 (0.80, 1.25)
Total T4	9.14 (7.13, 12.25)
TSH	1.87 (0.88, 3.50)
CRP	25.6 (6.35, 99.73)
Creatinine	0.90 (0.80, 1.10)
IL-6	9.92 (6.83, 28.88)

**Table 2:** Median (IQR) and comparison using the Kruskal Wallis test among T3, T4, TSH, and CRP values with COVID-19 severity TSH: thyroid-simulating hormone; CRP: C-reactive protein

	Mild	Moderate	Severe	H Value	P value
T3	1.22 (1.02, 1.50)	0.88 (0.80, 1.22)	0.911 (0.65, 1.11)	11.98	0.002
T4	9.90 (9.09, 14.11)	8.46 (6.22, 13.50)	8.25 (4.11, 11.68)	6.71	0.035
TSH	2.30 (0.85, 3.43)	2.30 (1.04, 4.11)	1.36 (0.88, 2.88)	1.30	0.521
CRP	10.50 (1.95, 29.35)	47.20 (6.50, 57.20)	95.22 (14.82, 156.37)	8.63	0.013

Severity of COVID-19 disease and thyroid function

We also found a clear influence of the severity of the disease on the effect on the thyroid, which was interestingly different from the findings of Zhang et al. and Lui et al. who found that the presence of thyroid dysfunction was not associated with more severe COVID-19 [17,27]. A statistically significant difference was seen in the values of T3 and T4 across the mild, moderate, and severe disease severity groups. T3 (H = 11.98, p =0.02) and T4 (H = 6.71, p = 0.035) were lower in patients with higher disease severity (Table 2). There was also a clear increase in the percentage of patients having low T3 while most patients had biochemically normal T4 and TSH (Table 3). The low T3 has been regarded as the “sick euthyroid syndrome,” “non-thyroidal illness syndrome (NTIS),” or “low T3 syndrome” [24]. In an early phase of the systemic disease, NTIS is an adaptive and protective state that conserves energy in an individual to cover for stress and being under macronutrient restriction [28].

**Table 3:** T3, T4, and TSH levels and severity of COVID-19 Reports of thyroid function tests in patients with different disease severities. TSH: thyroid-stimulating hormone

Total T3				
	MILD	MODERATE	SEVERE	TOTAL
DECREASED	1 (5%)	3 (15.7%)	9 (47.3%)	13 (22.8%)
NORMAL	18 (95%)	16 (84.2%)	10 (52.6 %)	44 (77.2%)
RAISED	0 (0%)	0 (0%)	0 (0 %)	0 (0%)
Total T4				
	MILD	MODERATE	SEVERE	
DECREASED	0 (0 %)	3 (15.7%)	3 (15.7%)	6 (10.5%)
NORMAL	13 (68.4 %)	11 (57.9%)	11 (57.9%)	35 (61.4%)
RAISED	6 (31.6 %)	5 (26.3%)	5 (26.3%)	16 (28.07%)
TSH				
	MILD	MODERATE	SEVERE	
DECREASED	2 (10.5 %)	0 (0 %)	3 (15.8%)	5 (8.7%)
NORMAL	15 (78.9 %)	16 (84.2 %)	16 (84.2 %)	47 (82.5%)
RAISED	2 (10.5 %)	3 (15.8%)	0 (0 %)	5 (8.7%)

A positive correlation was found between T3 and T4, which became more pronounced with increasing disease severity (Table 4); the correlation coefficient was highly increased between the mild and moderate groups while the increase was just minimal from moderate to severe, which can be explained by the decreased peripheral conversion of the thyroid hormone with an increase in disease severity, which eventually attains a plateau that can be attributed to the effect of cytokines and systemic inflammatory process on selenodeiodinases activity (especially D1) and abnormal production of thyroid-binding globulin (TBG) [25].

**Table 4:** Descriptive statistics and correlation matrix showing the variation in the correlation between the severity of COVID-19 disease (as per ICMR) and values of T3, T4, TSH, and CRP *. Correlation is significant at the 0.05 level (2-tailed) **. Correlation is significant at the 0.01 level (2-tailed) ICMR: Indian Council of Medical Research; CRP: C-reactive protein

MILD COVID DISEASE				
	T3	T4	TSH	CRP
T3	1.000			
T4	0.486*	1.000		
TSH	0.075	0.021	1.000	
CRP	-0.120	0.154	0.132*	1.000
MODERATE COVID DISEASE				
	T3	T4	TSH	CRP
T3	1.000			
T4	0.646^**^	1.000		
TSH	0.116	0.051	1.000	
CRP	-0.643	-0.750	-0.893^**^	1.000
SEVERE COVID DISEASE				
	T3	T4	TSH	CRP
T3	1.000			
T4	0.692^**^	1.000		
TSH	-0.189	-0.274	1.000	
CRP	0.037	0.131	-0.541^*^	1.000

Association of the bio-inflammatory marker, CRP, with TFT

A negative correlation was found between TSH and CRP (Table 3), which became more pronounced between mild and moderate disease severity while less pronounced between moderate and severe disease severity; this can be explained by two possible mechanisms. One mechanism involves a direct viral effect on the pituitary cells and the other involves an indirect effect in which the viral infection or its treatment produces systemic changes such as the activation of various proinflammatory cytokines [24,29]. There can be another probable direct effect causing decreased thyroid-releasing hormone gene expression in the hypothalamus as seen in other critical diseases [30]. The correlation between T3, T4, and CRP was not statistically significant.

Limitations

Being dependent on retrospective data, we couldn’t follow up with the patient for prognosis. The study design was a cross-sectional study so a comparison with the normal control group was missed, but since we already have literature on the effects of COVID and other viral diseases on thyroid function, the expected effects were compared, making three groups of disease severity, which was most feasible with the current study design.

Future prospects

Future studies are warranted to explore and confirm the molecular mechanisms behind the effects on thyroid function with increasing severity of COVID-19 and to explore the effects of other viral illnesses and the systematic inflammatory process produced by them on thyroid function.

## Conclusions

The purpose of the study was to study the effect of the acute phase of COVID-19 on thyroid function, assess the influence of the severity of the disease on thyroid function, and the association of these derangements with CRP. Most patients in the acute phase of COVID-19 had normal thyroid function. However, 47.3% of patients with severe disease showed low serum T3 levels. Furthermore, this study also indicated secondary hypothyroidism in around 16% of patients. Disease severity was seen to correlate with the extent of thyroid function abnormalities with severe disease having lower T3 values and normal to low TSH values. A significant negative correlation was seen between TSH and the bio-inflammatory marker, CRP. The findings of the study support the proposed mechanisms of the effect of COVID-19 systemic inflammatory process and direct viral effects on the thyroid gland, pituitary gland, and even peripheral conversion of thyroid hormones, all being affected in concordance with disease severity.
